# A method to isolate syncytiotrophoblast-derived medium-large extracellular vesicle small RNA from maternal plasma

**DOI:** 10.1016/j.placenta.2024.03.010

**Published:** 2025-06-13

**Authors:** William R. Cooke, Wei Zhang, Neva Kandzija, Gabriel Davis Jones, Christopher WG. Redman, Manu Vatish

**Affiliations:** Nuffield Department of Women's and Reproductive Health, University of Oxford, Level 3 Women's Centre, John Radcliffe Hospital, Oxford, OX3 9DU, UK

**Keywords:** Syncytiotrophoblast, Extracellular vesicles, RNA, Pregnancy, Preeclampsia

## Abstract

Syncytiotrophoblast-derived extracellular vesicles (STB-EVs) have an important role in placental research: both as mediators of feto-maternal signalling and as liquid biopsies reflecting placental health. Recent evidence highlights the importance of STB-EV RNA. Isolation of STB-EV RNA from maternal blood is therefore an important challenge. We describe a novel technique where we first separate medium-large particles from plasma using centrifugation then use a highly specific bead-bound antibody to placental alkaline phosphatase to separate STB-EVs from other similar-sized particles. We demonstrate the yield and size profile of small RNA obtained from plasma STB-EVs. We present data confirming isolation of placenta-derived micro RNA from maternal plasma using this method. The technique has been successfully applied to validate novel RNA discoveries from placental perfusion models. We propose it could offer new insights through transcriptomic analyses, providing a syncytiotrophoblast-specific signal from maternal blood.

## Highlights

• Circulating syncytiotrophoblast extracellular vesicles represent placental biopsies.
• Our method isolates syncytiotrophoblast extracellular vesicle RNA from maternal blood.
• We demonstrate small RNA yield, size profile and isolation efficacy.
• The method can validate findings from placental models and enable *in-vivo* discovery.

## Abbreviations

EV: Extracellular vesicle
PBS: Phosphate-buffered saline
PLAP: Placental alkaline phosphatase
RNA: Ribonucleic acid
STB-EV: Syncytiotrophoblast-derived extracellular vesicle

## 1 Introduction

In healthy pregnancy the human placenta releases extracellular vesicles (EVs) from the syncytiotrophoblast directly into the maternal circulation, in increasing quantities as the pregnancy progresses [1]. These EVs are decorated with proteins on their surface which direct them to specific maternal cells [2]. They are also filled with nucleic acids, lipids and proteins which can change distant cellular function. In placental diseases such as preeclampsia and gestational diabetes, the quantity and content of these EVs changes [3]. Syncytiotrophoblast-derived extracellular vesicles (STB-EVs) have been described as circulating biopsies reflecting placental health [4]. However, circulating STB-EVs are notoriously difficult to isolate. In blood, EVs are outnumbered by similar-sized particles such as lipoproteins and chylomicrons which frequently contaminate EV isolates [5]. Moreover, even at term, STB-EVs represent <1% of the circulating EV population [6].

**Fig. 1:**
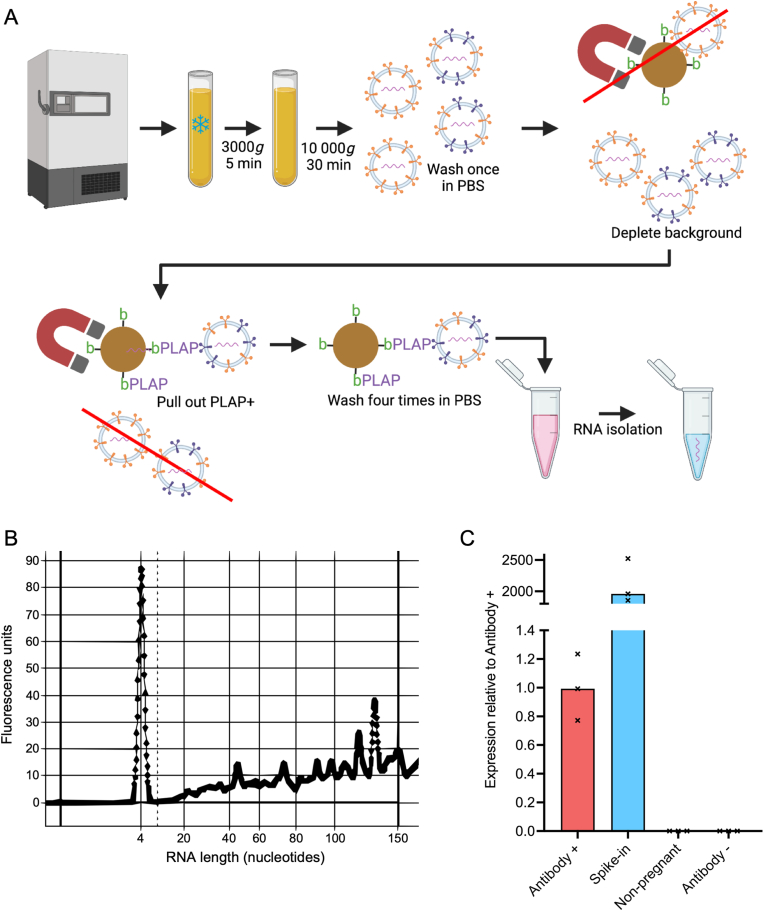
A: Diagram summarising key steps of the protocol for medium-large STB-EV RNA isolation from frozen maternal plasma (created with BioRender.com under licence).B: Representative electropherogram showing the size profile of small RNA isolated using the above technique. Abundance of small RNA molecules with different lengths is shown relative to a 4-nucleotide marker RNA.C: Relative expression of miR518b in four isolates (each n = 3) using the above technique. From left to right: healthy pregnant plasma using beads coated with NDOG2 (Antibody +); non-pregnant plasma spiked with STB-EVs from placental perfusion using beads coated with NDOG2 (Spike-in); non-pregnant plasma using beads coated with NDOG2 (Non-pregnant); healthy pregnant plasma using beads without NDOG2 but saturated with biotin (Antibody –). Expression normalised to spiked in cel-miR-39, displayed relative to Antibody +. Bars represent median.

Experimental studies use placental perfusion, explant culture or mechanical isolation to obtain and investigate STB-EVs [7]. Robust techniques are needed to isolate STB-EVs directly from maternal plasma: both to overcome the methodological shortcomings of experimental models, and to facilitate STB-EV interrogation as clinical biopsies. Numerous studies have used the syncytiotrophoblast-enriched protein placental alkaline phosphatase (PLAP) to quantify STB-EVs in the maternal circulation. The efficacy of these assays, often not empirically demonstrated, depends on antibody specificity. We found two reports of STB-EV isolation from blood in the literature; we could not replicate either from published methods [8,9]. We describe here a technique to first separate medium-large particles from plasma using centrifugation, then use a highly specific anti-PLAP antibody bound to magnetic beads to separate EVs of syncytiotrophoblast origin from others (Fig. 1A) [10]. We present data confirming enrichment of placenta-derived small RNA, providing strong confidence for the technique's efficacy.

## 2 Methods

### Ethical approval

Informed written consent should be obtained from participants, as in this study. Our project was approved by the Central Oxfordshire Research Ethics Committee (07/H0607/74 and 07/H0606/148).

### 2.1 Materials

Reagents and equipment are listed in Table 1.

**Table 1:** Summary of materials used in the described method. * is used to indicate where similar alternatives are considered unlikely to change the result.

Stage	Reagent/equipment	Manufacturer	Catalogue number
Plasma	***Equipment***		
	Eclipse™ Blood Collection Needle, 21 G x 1.25 inches	Becton, Dickinson and Company, NJ, USA	368650
	4.5 ml 3.2% sodium citrate vacutainer	Becton, Dickinson and Company, NJ, USA	369714
	Benchtop centrifuge	*Thermo Scientific, MA, USA	*75004515
	Micro Tubes suitable for cryogenic storage	*Sarstedt	*72.694.006
STB-EV	***Equipment***		
	Water bath	*Grant Instruments Ltd, Royston UK	*SAP5
	Temperature-controlled microcentrifuge	*Eppendorf, Hamburg, Germany	*5428000655
	Low protein binding microcentrifuge tubes	Eppendorf, Hamburg, Germany	10708704
	Balance (to 0.1 mg)	*Ohaus, Nanikon, Switzerland	*PX224M
	Vortex	*Scientific Industries Inc, NY, USA	*SI-0236
	Rotator	*Thermo Scientific, MA, USA	*88881001
	Magnet (Dynabeads™ MPC™-S)	Thermo Scientific, MA, USA	*A13346
	***Reagents***		
	NDOG2 antibody	Custom antibody	Available to collaborators
	Desalting columns	Thermo Scientific, MA, USA	89892
	Sulfo–NHS–LC-Biotin	Thermo Scientific, MA, USA	21335
	130 nm magnetic beads (MojoSort™ Streptavidin Nanobeads)	BioLegend, CA, USA	480016
	Biotin	Merck, NJ, USA	B4501
	Phosphate buffered saline (PBS)	*Sigma Aldrich, MO, USA	*D6537
RNA	***Equipment***		
	Phasemaker™ tube	Thermo Scientific, MA, USA	A33248
	Thermal cycler (QIAamplifier 96)	*Qiagen, Hilden, Germany	*9002991
	Agilent 2100 Bioanalyzer	Agilent Technologies, CA, USA	G2939BA
	***Reagents***		
	Bacteriophage MS2 carrier RNA solution	Roche, Basel, Switzerland	10165948001
	RNA spike-in (*5 nM cel-miR-39)	Integrated DNA Technologies, IO, USA	Sequence: 5′-UCACCGGGUGUA-AAUCAGCUUG
	TRIzol™ LS reagent	Thermo Scientific, MA, USA	10296010
	Chloroform (for molecular biology)	Thermo Scientific, MA, USA	J67241.AP
	RNAse-free glycogen	Roche, Basel, Switzerland	10901393001
	Isopropanol (for molecular biology)	Thermo Scientific, MA, USA	327272500
	Ethanol (for molecular biology)	Merck, NJ, USA	1085430250
	RNase-free-water	Merck, NJ, USA	W4502

*Tip 1: Low protein-binding microcentrifuge tubes should be used throughout. The authors compared four such commonly used tubes, finding Eppendorf's Protein LoBind® (Eppendorf, Hamburg, Germany) to be superior.

### 2.2 Prepare pregnant plasma

Collect whole blood using a 21-gauge needle directly into a citrate vacutainer, invert five times and process within 30 min. Spin at 1500 *g* for 15 min at room temperature.

Store supernatant (plasma) in 600 μL aliquots at −80 °C.

*Tip 2: Additional centrifugation steps to remove contaminating particles substantially reduces the signal from medium-large STB-EVs.

### 2.3 Biotinylate antibody

Pass 1.6 mg NDOG2 antibody through a desalting column to remove sodium azide preservative, dilute back to 1 mL volume with PBS. Add 12 μg Sulfo–NHS–LC-Biotin (as 5.5 mg/ml solution in ultrapure water) to the desalted antibody and incubate for 30 min. Pass the mixture through a second desalting column to remove excess biotinylation reagent, then dilute back to 1 mL volume with PBS.

*Tip 3: increasing antibody biotinylation above this level causes cross-links to form between magnetic beads when the antibody is added, resulting in bead clumping. This can be visualised both macroscopically and using Nanoparticle Tracking Analysis. We often encountered this problem during technique optimisation if commercially biotinylated antibodies were used.

### 2.4 Prepare biotin-saturated and NDOG2-coupled magnetic beads

Suspend 10 μL magnetic beads in 100 μL 0.3 μmolar biotin (solution in PBS), vortex and incubate at room temperature on rotator for 30 min. Separate the beads for 5 min on a magnet, and resuspend in 100 μL PBS.

*Tip 4: Before bead separation on a magnet, a pulse spin should always be used to remove solution from the tube lid.*

*Tip 5: Separation time is dependent on the magnet used and should be optimised.*

Suspend another 10 μL beads in 100 μL 6 ng/μL biotinylated NDOG2, incubate and separate as previously but resuspend in 100 μL 0.3 μmolar biotin for 30 min to saturate unbound streptavidin. Separate beads and resuspend in PBS as previously, to generate NDOG2-coupled beads ready for use. The above volumes should be scaled to the number of samples being processed, adding 10% for pipetting losses.

### 2.5 Isolate STB-EVs from pregnant plasma

Thaw 600 μL plasma aliquot briefly in a water bath at 37 °C, invert five times and immediately spin at 3000 *g* for 5 min at 4 °C to remove cryoprecipitates. Spin 500 μL supernatant at 10,000 *g* for 30 min at 4 °C to pellet medium-large particles. Remove 450 μL supernatant, resuspend pellet in 450 μL PBS, spin a second time with the same settings. Remove 450 μL supernatant, add 100 μL PBS and resuspend pellet. Add 100 μL biotin-saturated beads to deplete non-specific binding, vortex and incubate at room temperature on rotator for 30 min. Separate beads for 5 min on a magnet, and add supernatant to 100 μL NDOG2-coupled beads. Incubate at room temperature on rotator for 30 min. Separate beads for 5 min on a magnet, resuspending them in 250 μL PBS to wash. Repeat four times, then resuspend beads in 250 μL PBS and transfer to Phasemaker™ tube.

### 2.6 Isolate RNA

Add 2.5 μL bacteriophage carrier RNA, 1 μL RNA spike-in and 750 μL TRIzol™ LS to Phasemaker™. Isolate RNA following manufacturer's instructions, with the addition of 1 μL RNase-free glycogen to the aqueous phase before adding isopropanol. Resuspend the RNA pellet in 60 μL of RNase-free water. Confirm the quantity and size of isolated RNA using the BioAnalyzer. Store at −80 °C.

*Tip 6: Qualification of RNA is not possible: ribosomal RNA subunits are typically absent from circulating EVs, preventing calculation of RNA integrity number; phenol contamination with low abundance of RNA renders 260 nm *spectrophotometry (e.g. Nanodrop™) invalid* [11].

### 2.7 Validation

To demonstrate isolation efficacy, miR518b was quantified using TaqMan™ MicroRNA Assay #001156, normalised to cel-miR-39 spike-in (#000200) and relative expression calculated using the Pfaffl approach [12].

## 3 Results and discussion

See Fig 1A for a diagram of this technique. For evaluation of RNA yield, the method was applied to six healthy third trimester plasma samples with mean gestational age 30 + 6 weeks (range 27 + 6 to 33 + 1 weeks). 500 μL plasma yielded mean 61 ng small RNA species sized 0–150 nucleotides from STB-EVs (range 37–85 ng). 21% of these species were of length 10–40 nucleotides, containing signal from micro RNAs and tRNA fragments (range 18–22%). A representative electropherogram showing the relative abundance of small RNA species of different lengths is shown in Fig 1B.

To demonstrate isolation efficacy, we used a placenta-specific micro RNA from the chromosome 19 microcluster (miR518b); selecting a placenta-specific target overcomes potential confounding from multiple other sources of RNA within plasma [13]. miR518b was quantified in third-trimester healthy pregnant plasma (Fig. 1C, Antibody +) and compared to three controls. The positive control (Fig. 1C, Spike-in) represented non-pregnant plasma with perfusion-derived medium-large STB-EVs added at 2.4E10 particles/ml plasma. The negative controls were non-pregnant plasma from female volunteers of reproductive age (Fig 1C, Non-pregnant) and healthy pregnant plasma interrogated with biotin-saturated beads (Fig. 1C, Antibody –). Beads were not saturated in third-trimester plasma: miR518b was approximately 2000-fold more abundant in the positive control. miR518b was not detected in the Antibody – control, confirming the method's specificity.

We have employed this technique to validate disease-specific differences in STB-EV tRNA fragment expression identified using placental perfusion [14]. We propose it could offer the opportunity to investigate the *in-vivo* validity of other discoveries from *ex-vivo* STB-EV models, including placental explants. The technique could also be used in hypothesis-generating experiments, enabling direct *in-vivo* assessment of the medium-large STB-EV transcriptome in health and disease.

We found only two published methods for the isolation of STB-EVs from maternal blood. Srinivasan and colleagues incubated serum with biotinylated PLAP antibody, then directly added 1 μm streptavidin magnetic beads [8]. This approach overlooks soluble PLAP, which exceeds EV-bound PLAP by orders of magnitude and would confound the STB-EV isolate [15]. Lai and colleagues used differential ultracentrifugation to separate small EVs from plasma, adding them to agarose beads pre-incubated with anti-PLAP antibody [9]. They did not specify the provenance of most materials, including beads or antibody; it is therefore not possible to replicate their findings. Additionally, the authors validated their PLAP isolation with Western blots using the same unspecified antibody; this fails to confirm the antibody's specificity.

Our technique's strength lies in the selectivity of the monoclonal antibody, NDOG2, which we made in our department and validated across 14 different human tissues [10]. By employing magnetic beads with a similar diameter to the particles being bound, we maximise steric interactions between STB-EVs and bead-bound antibody. Finally, we validate our method using a placenta-specific micro RNA – a different approach from that used for isolation (an antibody to a placenta-enriched protein). The technique is limited by the number of bead separations required, making it time-consuming and low-throughput in the laboratory. Nevertheless, it provides accurate quantitation, which has enabled successful validation of findings from placental perfusion studies *in-vivo* [14].

In summary, we present and validate a novel and sensitive technique to interrogate small RNA within STB-EVs isolated from maternal plasma.

## CRediT authorship contribution statement

**William R. Cooke:** Writing – review & editing, Writing – original draft, Visualization, Validation, Software, Resources, Project administration, Methodology, Investigation, Funding acquisition, Formal analysis, Data curation, Conceptualization. **Wei Zhang:** Writing – review & editing, Supervision, Methodology, Conceptualization. **Neva Kandzija:** Writing – review & editing, Validation, Methodology. **Gabriel Davis Jones:** Writing – review & editing, Visualization, Methodology. **Christopher WG. Redman:** Writing – review & editing, Supervision, Methodology, Investigation, Conceptualization. **Manu Vatish:** Writing – review & editing, Supervision, Methodology.

## Declaration of competing interest

The authors declare the following financial interests/personal relationships which may be considered as potential competing interests:

William Cooke reports financial support was provided by 10.13039/100010269Wellcome Trust. If there are other authors, they declare that they have no known competing financial interests or personal relationships that could have appeared to influence the work reported in this paper.
